# Impact of Timing of Endoscopy on Mortality in Non-variceal Upper Gastrointestinal Bleeding: A Retrospective Cohort Study -

**DOI:** 10.31661/gmj.vi.3550

**Published:** 2025-06-29

**Authors:** Mahdi Foroughian, Ali Ravaghi, Seyed Reza Habibzadeh, Ladan Goshayeshi, Zahra Abbasi Shaye, Maryam Ziyaei, Morteza Talebi Doluee

**Affiliations:** ^1^ Department of Emergency Medicine, Faculty of Medicine, Mashhad University of Medical Sciences, Mashhad, Iran; ^2^ Department of Emergency Medicine, Mashhad University of Medical Sciences, Mashhad, Iran; ^3^ Department of Gastroenterology and Hepatology, Faculty of Medicine, Mashhad University of Medical Sciences, Mashhad, Iran; ^4^ Department of Community Medicine, Faculty of Medicine, Mashhad University of Medical Sciences, Mashhad, Iran; ^5^ Department of Emergency Medicine, Faculty of Medicine, Zahedan University of Medical Sciences, Zahedan, Iran

**Keywords:** Mortality, Gastrointestinal Bleeding, Endoscopy

## Abstract

**Background:**

Non-variceal upper gastrointestinal bleeding is a common problem worldwide, which is associated with a significant mortality rate. The purpose of this study was to investigate the relationship between the mortality rate of patients referred to the emergency room with non-variceal upper gastrointestinal bleeding and the time of therapeutic-diagnostic endoscopy.

**Materials and Methods:**

This study was a retrospective cohort observational study at Imam Reza Hospital in Mashhad, which was conducted in patients presenting with obvious symptoms of non-variceal acute gastrointestinal bleeding between April 2017 and March 2018. Underlying variables, endoscopic history, hemoglobin level, Glasgow – Blatchford score, blood pressure and the endoscopic result were extracted from patients’ records. The time of death was followed up by telephone within 30 days after hospitalization. Data were compared based on the time of endoscopy since arrival. Patients with gastrointestinal bleeding were initially evaluated in the emergency room, unstable patients were transferred to the emergency room for stabilization and initial measures, and other patients were transferred to the emergency room, and the unstable patients were excluded from the plan.

**Results:**

In this study, 189 patients (with an average age of 60.11 ± 17.59 years) were examined. 23 cases (12.16%) of death were recorded within 30 days. 26 people (13.75%) underwent emergency endoscopy within 0 to 6 hours of referral. Forty-four people (23.28%) underwent endoscopy within 6 to 12 hours and the rest (119 people, 62.96%) within 12 to 24 hours. There was no significant difference between deceased and recovered subjects in terms of various study variables, including Blatchford score, number of days hospitalized in the ward and intensive care unit, and the number of units of compressed red blood cells injected (P0.05). Diabetes was significantly more prevalent in patients undergoing endoscopy 12 h compared to the 12 h (3.36% vs. 32.86%; P=0.001). adjusting for diabetes, the timing of endoscopy (within 12 hours vs. after 12 hours) was not significantly associated with mortality, with both crude (OR 1.25, 95% CI 0.63-2.49, P=0.523) and adjusted (OR 1.30, 95% CI 0.65-2.60, P=0.456) odds ratios.

**Conclusion:**

Our study showed no association between endoscopy time and mortality in patients with upper gastrointestinal bleeding; however, this finding should be confirmed in future studies in more controlled populations as a clinical trial.

## Introduction

Gastrointestinal bleeding (GIB) has a significant impact on mortality. Bleeding of the upper gastrointestinal tract is caused by injury and local damage that causes ulceration of the mucosa of the gastrointestinal tract [1][2]. Gastrointestinal bleeding (GIB) is divided into two categories with upper and lower origin. Bleeding that occurs in the upper part of the duodenum is called upper gastrointestinal bleeding (UGIB). If the bleeding is from the duodenum to the anus, it is called lower gastrointestinal bleeding (LGIB) [3]. GIB, particularly UGIB, is a significant medical concern with notable epidemiological patterns and mortality rates [4]. The mortality rate among patients experiencing acute upper gastrointestinal bleeding ranges from 3% to 14%, with specific risk factors influencing outcomes [5]. Rebleeding is particularly high in cases of variceal and peptic ulcer bleeding, contributing to the complexity of managing these conditions [6]. Epidemiological studies indicate variations in UGIB-related mortality, with rates ranging from 0.9 to 9.8 per 100,000 person-years. Additionally, mortality from upper GI bleeding shows age-related trends, with rates at 0.4% in patients under the age of 60 and increasing to 11% in those over the age of 80 [7]. Untreated aortic intestinal fistulas presenting with upper gastrointestinal bleeding exhibit close to 100% mortality, underscoring the critical nature of certain underlying conditions [8]. The global epidemiology of both upper and lower gastrointestinal bleeding reveals varying mortality rates, emphasizing the need for comprehensive understanding and tailored management strategies [8]. Non-Variceal Upper Gastrointestinal Bleeding (NVUGIB) is defined as bleeding that occurs in the esophagus, stomach, or proximal duodenum and is not associated with the presence of varices, which are enlarged and swollen veins [9]. It is a significant medical condition that can lead to complications if not promptly diagnosed and treated. The symptoms of NVUGIB may include hematemesis (vomiting of blood), melena (black, tarry stools), and signs of hemorrhagic shock [10]. Patients with NVUGIB might also present with coffee ground vomiting, indicating partially digested blood. These symptoms often signify active bleeding in the upper gastrointestinal tract and warrant urgent medical attention. It's essential to distinguish between variceal and non-variceal bleeding as the management strategies can differ. Prompt diagnosis through endoscopy and appropriate therapeutic interventions are crucial in the effective management of Non-Variceal Upper Gastrointestinal Bleeding [10]. NVUGIB is generally more common than Variceal Upper Gastrointestinal Bleeding (VUGIB) [11]. The incidence of acute upper gastrointestinal bleeding, particularly nonvariceal cases, has shown an increasing trend during the COVID-19 pandemic [12]. Mortality rates for NVUGIB can vary, with reported rates ranging from 3% to 12%, emphasizing the need for a nuanced understanding of the factors influencing outcomes in these cases [13]. Recent studies have focused on validating risk score systems for non-variceal upper gastrointestinal bleeding, aiming to improve prognostic accuracy and guide appropriate interventions [14][15]. Understanding the epidemiological data on acute gastrointestinal bleeding, particularly in the non-variceal context, is crucial for informed medical management and improved patient outcomes [16][17]. The timing of endoscopy in NVUGIB has been a topic of investigation. Studies, such as the ones cited, have explored the impact of early endoscopy on clinical outcomes [18]. The results have shown conflicting findings, with some studies suggesting no significant difference in mortality rates between patients who received early versus nonearly endoscopy in cases of high-risk bleeding [10]. Additionally, data on the comparison of urgent (within 12 hours) and early endoscopy (within 24 hours) in NVUGIB have presented conflicting results, highlighting the ongoing debate on the optimal timing of endoscopy in these cases [19][20]. The controversy continues, and it is essential to consider individual patient factors and the specifics of the bleeding episode when determining the optimal timing for endoscopy. Studies have also explored the impact of upper endoscopy on patients with upper gastrointestinal hemorrhage, emphasizing that the timing of endoscopy can influence outcomes in cases of acute NVUGIB [21]. However, the optimal timing remains a matter of debate within the medical community [22]. Given that early endoscopy of the upper gastrointestinal tract within 24 hours following the onset of NVUGIB enhances both therapeutic and diagnostic outcomes for patients, a comprehensive examination of the effects of endoscopy at specific time intervals—0-6 hours, 6-12 hours, and 12-24 hours post-referral—among individuals experiencing NVUGIB is of paramount significance. The purpose of this study was to investigate the relationship between the mortality rate of patients referred to the emergency room with non-variceal upper gastrointestinal bleeding and the time of therapeutic-diagnostic endoscopy.

## Materials and Methods

The present investigation constitutes a retrospective cohort observational study devoid of intervention. The study encompassed patients referred to the Adalatian Emergency Department of Imam Reza Hospital (PBUH) exhibiting apparent symptoms of acute upper gastrointestinal bleeding (hematosis, melena, or both). The study period extended from April 2017 to March 2018, and a total of 189 eligible patients were recruited for inclusion. Criteria for inclusion comprised a Glasgow-Blatchford score of 12 or higher, age exceeding 18 years, non-pregnancy, absence of shock, and stability of the patient's veins in the emergency room. Exclusion criteria encompassed patient unwillingness to participate, underlying coagulation disorders, unstable conditions in the emergency room, and failure to complete follow-up.

Patients were categorized based on the Glasgow-Blatchford score of 12 or higher, indicating a high risk of major bleeding or death. This scoring system, initially introduced by Hadzibulic et al. [23], stratified patients into three groups based on the time of endoscopy: first 6 hours, 6-12 hours, and 12-24 hours.

The Blatchford system, acknowledged for clinical decision-making and efficient monitoring, was employed to evaluate patients with acute upper gastrointestinal bleeding [24]. This system assessed risk based on clinical variables, including increased urea level, decreased hemoglobin level, decreased systolic blood pressure, rapid pulse, syncope, and the presence of melena. Patients with a score above 5 were considered high-risk for endoscopic treatment, while those with a score below 4 did not require immediate endoscopic intervention.

Patients admitted to the ward were scrutinized in three-time categories (0-6 hours, 6-12 hours, and 12-24 hours) based on their endoscopy time, resulting in five categories: clean base, Flat Spot, Clot, Visible Vessel, and Active Bleeding. The primary objective of the study was to assess the 30-day mortality of patients who underwent endoscopy within these specified time periods. Patient follow-up occurred either in the clinic or via phone if in-person follow-up was not feasible.

Demographic information, including age, sex, underlying diseases, history of endoscopy, peptic ulcer history, NSAID drug usage, aspirin, clopidogrel, anticoagulant drug usage, and clinical details such as type of symptoms, blood pressure on arrival, heart rate, bleeding in hospital at the beginning of hospitalization, Glasgow-Blatchford score, endoscopy result, re-bleeding during hospitalization, and the need for a blood cell pack, were meticulously recorded in the checklist.

### Ethical Considerations

This research adheres to stringent ethical standards, having obtained approval from the ethics committee of Mashhad University of Medical Sciences. Throughout the study, all stages were meticulously conducted in accordance with the specified ethical protocols. In an effort to safeguard the privacy and anonymity of the research subjects, their names and surnames were deliberately excluded from the checklist. Moreover, robust measures were implemented to ensure the confidentiality of all information derived from the research community. The ethical approval, granted with the code IR.MUMS.MEDICAL.REC.1400.072, underscores the commitment to ethical principles.

### Statistical Analysis

In terms of statistical methodology, qualitative variables were effectively represented using frequency and percentage, while mean and standard deviation served as expressions for quantitative variables. Rigorous checks for data normality were conducted employing the Kolmogorov-Smirnov test. Comparative analyses of both qualitative and quantitative variables across different endoscopy times, inclusive of general and endoscopy result-based comparisons, were carried out through a combination of Chi-Square, independent T tests, ANOVA, or their non-parametric counterparts. The Chi-square statistic was specifically employed to scrutinize qualitative results among three distinct times of endoscopy. Multivariate logistic regression was performed for adjusting for factors that were significant in univariable analysis. A predetermined significance level of P-value<0.05 was considered. The statistical analysis utilized SPSS version 16 software (IBM Corporation, Armonk, NY, USA), with regression incorporated when deemed necessary for controlling confounding factors.

## Results

**Table:** Table1. Clinical Characteristics of Patients with Non-variceal Upper Gastrointestinal Bleeding Based on Prognosis

		**Recovered** **(N=143)**	**Deceased (N=23) **	**P-value**
**Age, year, median (Q1 - Q3)**		67 (55-71)	62 (44-76)	0.425
**Gender, N (%)**	**Male**	105(73.43%)	16(69.57%)	0.699
	**Female**	38(26.57%)	7(30.43%)	
**Hemoglobin, mg/dL, median (Q1 - Q3)**		‎9.7(7.5-11.8)‎	‎8.5(7.4-11.9)	0.297
**Number of units of packed red blood cells injected, median (Q1 - Q3) **		‎1(0-2)	‎2(0-4)	0.236
**Systolic blood pressure, mm Hg, median (Q1 - Q3)**		120 (100-134)	108 (100-147)	0.606
**Diastolic blood pressure, mm Hg, median (Q1 - Q3)**		80 (69-80)	80 (60-89)	0.983
**History of previous gastrointestinal bleeding, number (%) **		0(0%)	13(56.52%)	0.218
**Blatchford score, median (Q1 - Q3)**		10 (7-12)	10 (8-13)	0.298
**Number of days hospitalized in ICU, median (Q1 - Q3)**		5 (3-7)	5 (3-10)	0.135
**Number of hospitalization days (mean ± SD)**		0.39±1.07	6.2±2.04	0.034
**Endoscopy in < 6 hours, N (%)**		19(13.29%)	3(13.04%)	
**Endoscopy in < 12 hours, N (%)**		52(36.36%)	10(43.48%)	0.793
**Endoscopy after 12 hours, N (%)**		91(63.64%)	13(56.52%)	
	**Hernia Hiatal**	19(13.29%)	3(13.04%)	0.651
	**visible vessel**	52(36.36%)	10(43.48%)	0.623
	**Active bleeding**	91(63.64%)	13(56.52%)	0.25
**Pathophysiology based on endoscopic findings, N (%) **	**Adherent**	9(6.29%)	2(8.7%)	0.762
	**clean base**	4(2.8%)	0(0%)	0.369
	**normal**	5(3.5%)	2(8.7%)	0.978
	**Mallory-Weiss**	3(2.1%)	0(0%)	0.762
	**other**	66(46.15%)	8(34.78%)	0.198

**Table:** Table2. Clinical Characteristics of Patients with Non-variceal Upper Gastrointestinal Bleeding Based on Time of Endoscopy

		**>12 h** **(n=119)**	**<12 h** **(N=70)**	**P-value**
		**N (%) **	**N (%)**	
**Gender **	**Male **	84(70.59%)	52(74.29%)	0.564
	**Female**	35(29.41%)	18(25.71%)	
**History of gastrointestinal bleeding **		9(7.56%)	7(10%)	0.714
	**No underlying disease**	30(25.21%)	20(28.57%)	0.652
	**Heart disease **	9(7.56%)	6(8.57%)	0.751
	**blood pressure**	9(7.56%)	5(7.14%)	0.787
	**diabetes**	4(3.36%)	23(32.86%)	0.001
**Underlying disease**	**liver disease**	1(0.84%)	2(2.86%)	0.695
	**Heart disease with other diseases **	27(22.69%)	12(17.14%)	0.093
	**Blood pressure with other diseases**	11(9.24%)	9(12.86%)	0.726
	**Diabetes with other diseases **	2(1.68%)	1(1.43%)	0.698
	**other**	26(21.85%)	13(18.57%)	0.294
**Endoscopic findings**	**Hernia Hiatal **	7(5.88%)	4(5.71%)	0.759
	**visible vessel**	4(3.36%)	1(1.43%)	0.692
	**Active bleeding **	5(4.2%)	3(4.29%)	0.753
	**Adherent**	1(0.84%)	2(2.86%)	0.697
	**clean base **	46(38.66%)	39(55.71%)	0.291
	**normal**	21(17.65%)	9(12.86%)	0.295
	**Mallory-Weiss**	3(2.52%)	1(1.43%)	0.694
	**other**	32(26.89%)	11(15.71%)	0.098

**Table:** Table3. Regression Analysis of the Effect of Endoscopy Timing on Mortality, Adjusted for Diabetes

**Variable**	**Crude OR (95% CI)**	**P-value**	**Adjusted OR (95% CI)**	**P-value**
**Endoscopy Timing**				
**12 hours (Ref)**	1.00	-	1.00	-
**> 12 hours**	1.25 (0.63 - 2.49)	0.523	1.30 (0.65 - 2.6)	0.456

In this study, a total of 189 patients underwent examination, with 136 (72%) being male and 53 (28%) females. The average age of the subjects was 60.11 ± 17.59 years. Notably, 16 individuals (8.5%) had a previous history of Gastrointestinal Bleeding (GIB), while 50 people (26.5%) had no prior diseases. Clinical examination findings at the beginning of hospitalization revealed an average systolic blood pressure of 120.93±21.72 mmHg and an average diastolic blood pressure of 76.12±16.40 mmHg. The initial venous blood test displayed an average hemoglobin level of 9.82±3.07 mg/dL. Blatchford's average score was 9.41±3.94. The subjects were hospitalized for an average of 6.44 ± 6.37 days, with an average of 0.60 ± 2.66 days spent in the intensive care unit. Patients received an average of 1.65 ± 2.09 units of packed red blood cells. Regarding the diagnostic process, 63% of patients underwent endoscopy between 12 and 24 hours after the request. The most common diagnostic finding was a clean base ulcer, reported in 45% of participants. Mallory-Weiss was observed in only 4 cases (1.2%), and 30 people (15.9%) had a normal endoscopic result. Examining the clinical course, the final outcome was categorized as complete recovery or death, with 23 cases (12.16%) resulting in death. Another 23 patients (12.16%) were excluded due to a lack of follow-up and registration of the final outcome.

Table-1 delineates key demographic and clinical features of patients, stratified by outcomes - either deceased (N=23) or recovered (N=143). The median age for the deceased was noted at 67 years (Q1 - Q3: 55-71), slightly higher than the recovered group, which had a median age of 62 years (Q1 - Q3: 44-76) (P=0.425). The gender distribution indicated 16 males (69%) and 7 females (30%) in the deceased group, whereas the recovered group comprised 105 males (73%) and 38 females (26%) (P=0.699). Examining hemoglobin levels in mg/dL, the study revealed a median of 5.8 (Q1 - Q3: 9.11-4.7) in the deceased group and 7.9 (Q1 - Q3: 8.11-5.7) in the recovered group, showing a non-significant difference (P=0.297). The median number of units of packed red blood cells administered was 2 (Q1 - Q3: 0-4) for the deceased and 1 (Q1 - Q3: 0-2) for the recovered (P=0.236). Systolic blood pressure exhibited a median of 108 mm Hg (Q1 - Q3: 100-147) for the deceased and 120 mm Hg (Q1 - Q3: 100-134) for the recovered, displaying no significant contrast (P=0.606). Diastolic blood pressure had a median of 80 mm Hg (Q1 - Q3: 60-89) for the deceased and 80 mm Hg (Q1 - Q3: 69-80) for the recovered, with no significant distinction (P=0.983). A minimal percentage (1.9%) of the deceased had a history of previous gastrointestinal bleeding, compared to none in the recovered group (P=0.218). The Blatchford score median was 10 (Q1 - Q3: 8-13) for both the deceased and recovered, revealing no significant difference (P=0.298). The number of days hospitalized in the ICU showed a median of 5 (Q1 - Q3: 3-10) for both groups. The mean number of hospitalization days was 6.2 ± 2.04 for the deceased and 0.39 ± 1.07 for the recovered, demonstrating a significant contrast (P=0.034). Regarding endoscopy timing, 13% of the deceased underwent endoscopy in less than 6 hours, while 28.13% of the recovered group had endoscopy within the same timeframe (P=0.793). Further categorization revealed differences in the proportion of individuals who had endoscopy within 12 hours (47.43% deceased vs. 36.36% recovered, P=0.651) and after 12 hours (53.56% deceased vs. 64.63% recovered, P=0.623, Table-2). The clinical characteristics of patients with non-variceal upper gastrointestinal bleeding based on the timing of endoscopy (within 12 hours vs. after 12 hours) reveal several notable differences and similarities. The gender distribution was similar between the two groups, with a slight majority of males in both (74.29% in the <12 h group and 70.59% in the >12 h group). The history of gastrointestinal bleeding and the presence of underlying diseases, including heart disease, blood pressure issues, liver disease, and other conditions, showed no significant differences between the groups. However, diabetes was significantly more prevalent in the <12 h group (32.86%) compared to the >12 h group (3.36%), with a P-value of 0.001. Endoscopic findings also showed some variations: a clean base was more common in the <12 h group (55.71%) compared to the >12 h group (38.66%), although this difference was not statistically significant. Conversely, the >12 h group had a higher proportion of other findings (26.89%) compared to the <12 h group (15.71%), though this difference also did not reach statistical significance (Table-2). In the multivariate logistic regression analysis adjusting for diabetes history, the timing of endoscopy (within 12 hours vs. after 12 hours) was not significantly associated with mortality, with both crude (OR 1.25, 95% CI 0.63-2.49, P=0.523) and adjusted (OR 1.30, 95% CI 0.65-2.60, P=0.456) odds ratios failing to reach statistical significance (Table-3).

## Discussion

The primary objective of this study was to explore the association between the mortality rate of patients presenting with non-variceal upper gastrointestinal bleeding in the emergency room and the timing of therapeutic-diagnostic endoscopy. The study focused on two key outcomes: mortality and the time of endoscopy. Out of the examined individuals, 23 cases (12.16%) resulted in mortality. An additional 23 patients (12.16%) were excluded from the mortality analysis due to insufficient follow-up and the absence of recorded outcomes of death. However, it's important to note that the information regarding these excluded individuals was still considered in the investigation of the endoscopy time. This dual focus on mortality and endoscopy time contributes to a comprehensive understanding of the factors influencing patient outcomes in the context of non-variceal upper gastrointestinal bleeding.

The average death rate in individuals with upper gastrointestinal bleeding is approximately 10%, aligning with our study's finding of 12.16%, consistent with prior research [4][25][26]. When investigating potential risk factors distinguishing deceased from recovered individuals, our study found no correlation between demographic factors such as age, gender, or history of previous diseases and mortality. This aligns with Moldina et al.'s retrospective cohort study, where demographic variables showed no correlation with mortality, though the type of bleeding disease was a factor in Moldina et al.'s study, impacting mortality [26]. Our study excluded individuals with esophageal varices, differing from Moldina et al.'s study, where esophageal varices were the most common cause of upper gastrointestinal bleeding. The overall mortality rate in their study was notably higher at 33.5%, emphasizing the influence of underlying conditions. In our study, mortality was comparable to global rates [27], potentially attributed to late presentation or high severity of bleeding. Emergency endoscopy timing varied in our study, with 13.75% within 0-6 hours, 23.28% within 6-12 hours, and 62.96% within 12-24 hours. No significant association between emergency endoscopy and one-month mortality was observed. Contrary to Guo et al.'s findings, our results did not support a lower mortality rate with early endoscopy. Chu et al. emphasized urgent endoscopy as an independent predictor of mortality but not rebleeding. Lau et al. similarly found no significant relationship between mortality and endoscopy timing, reinforcing our study's consistency with existing literature [28][29][30][31].

The current study stands out for its innovative approach, addressing a relatively novel idea with limited similar studies in the field. However, it is important to acknowledge certain limitations. The study's sample size is relatively smaller compared to similar research, attributed to the smaller scale of the medical centers involved, reflecting the single-center nature of the study. The smaller sample size warrants caution in generalizing findings to broader populations. Additionally, the retrospective design introduces potential hidden confounding variables, emphasizing the need for future clinical trials to validate the results. Conducting a meta-analysis could provide a more comprehensive understanding, but existing meta-analyses have focused on a narrower time frame (24 hours before or after the visit), deviating from the hypothesis of very urgent endoscopy within 6 hours. To enhance the study's scope, future investigations could explore additional indicators beyond those considered in this study. For instance, Ren et al.'s [32] inclusion of parameters like the time for fecal occult blood to turn negative and the recovery of bowel sounds could offer a more nuanced evaluation. Incorporating these aspects into research protocols would contribute to a more comprehensive understanding of the outcomes associated with early and delayed endoscopy.

## Conclusion

The present study found that emergency endoscopy done within 6 hours, between 6 and 12 hours, or as an elective treatment beyond 12 hours did not significantly affect patient mortality. Patients got the same quantity of packed red blood cell transfusions regardless of endoscopic scheduling. These findings suggest that emergency endoscopic mortality and transfusion needs are constant across periods. These findings suggest that endoscopy scheduling may not significantly affect death rates or transfusion demands in non-variceal upper gastrointestinal hemorrhage.

## Conflict of Interest

The authors declare that there is no conflict of interest associated with this research.
